# Historical and Molecular Perspectives on the Presence of *Helicobacter pylori* in Latin America: A Niche to Improve Gastric Cancer Risk Assessment

**DOI:** 10.3390/ijms25031761

**Published:** 2024-02-01

**Authors:** Roxana González-Stegmaier, Patricia Aguila-Torres, Franz Villarroel-Espíndola

**Affiliations:** 1 Traslational Medicine Laboratory, Instituto Oncológico Fundación Arturo López Pérez, Santiago 7500000, Chile; roxana.gonzalez@falp.org; 2 Laboratorio de Microbiología Molecular, Escuela de Tecnología Médica, Universidad Austral de Chile, Puerto Montt 5480000, Chile; patricia.aguila@uach.cl

**Keywords:** gastric cancer, Latin American, *Helicobacter pylori*, cagA, vacA, Chile

## Abstract

*Helicobacter pylori* (*H. pylori*) is responsible for causing chronic gastritis, which can cause peptic ulcer and premalignant lesions such as atrophic gastritis, intestinal metaplasia, and dysplasia, with the risk of developing gastric cancer. Recent data describe that *H. pylori* colonizes the gastric mucosa of more than 50% of the world’s population; however, this bacterium has been described as infecting the human population since its prehistory. This review focuses on the populations and subpopulations of *H. pylori*, differentiated by the polymorphisms present in their constitutive and virulence genes. These genes have spread and associated with different human populations, showing variability depending on their geographical distribution, and have evolved together with the human being. The predominant genotypes worldwide, Latin America and Chile, are described to understand the genetic diversity and pathogenicity of *H. pylori* in different populations and geographic regions. The high similarity in the sequence of virulence genes between *H. pylori* strains present in Peruvian and Spanish natives in Latin America suggests a European influence. The presence of cagA-positive strains and vacA s1 m1 allelic variants is observed with greater prevalence in Chilean patients with more severe gastrointestinal diseases and is associated with its geographical distribution. These findings highlight the importance of understanding the genetic diversity of *H. pylori* in different regions of the world for a more accurate assessment of the risk of associated diseases and their potential impact on health.

## 1. Introduction

*H. pylori* is a Gram-negative, microaerophilic bacterium that colonizes the gastric mucosa of more than 50% of the world’s population [1]. In 1994, the International Agency for Research on Cancer classified it as a Group I carcinogen [2,3]. Infection can be acquired in childhood and persist asymptomatically throughout life [4]. *H. pylori* causes chronic gastritis. This can lead to peptic ulcers as well as premalignant lesions such as atrophic gastritis, intestinal metaplasia, and dysplasia, which can eventually trigger gastric cancer [5]. Atrophic gastritis is the first step in the premalignant cascade and is characterized by the loss of gastric glandular cells, resulting in reduced acid production and an increase in pH levels [6,7]. Intestinal metaplasia refers to the transformation of the gastric epithelium into an intestinal-type epithelium, which can be complete or incomplete, depending on the presence of goblet cells among the epithelial cells [6,8]. Dysplasia is characterized by a neoplastic phenotype, both in terms of cell morphology and organization. In this state, the epithelium shows enlarged, hyperchromatic, and stacked nuclei. The cells no longer remain within the boundaries of the basal membrane, and the architecture of the glands becomes irregular, occasionally bifurcated or branched, and may even develop pseudopapillae [6]. All these changes can present a gradual transformation from well-differentiated to poorly differentiated and are classified as low or high grade, reflecting the cancer risk associated with each phenotype [6].

Furthermore, *H. pylori* possesses several virulence factors, among which the cytotoxin-associated gene A (*cagA*) and the vacuolating cytotoxin (*vacA*) are the most important. These are polymorphic and exert multiple effects on human cells [9]. The CagA protein is one of the products encoded by the cag pathogenicity island (*cagPAI*) and is translocated into human epithelial cells during infection with *H. pylori* through the type IV secretion system [10]. Within the host cells, CagA is phosphorylated by Src family kinases at the tyrosine of the EPIYA motif (Glu-Pro-Ile-Tyr-Ala) [11,12]. Based on amino acid sequences, EPIYA motifs can be classified into EPIYA-A, -B, -C, or -D [9]. EPIYA-A, -B, and a variable number of -C are generally found in clinical strains isolated from Western populations, while EPIYA-A, -B, and -D are found in strains from East Asia [13]. Infection with CagA-positive *H. pylori* strains significantly increases the risk of gastric cancer as these present higher degrees of gastric inflammation and epithelial cell damage compared to individuals from whom CagA-negative strains have been isolated [14,15]. The prevalence of CagA-positive strains varies between ethnic groups and regions; in Asia, prevalence reaches over 90% compared to 50–60% in Western countries [16].

The VacA protein is an intracellular action exotoxin that affects multiple cellular pathways in different types of host cells, induces vacuolization, and causes cell death in the host cell [17]. Although nearly all *H. pylori* strains isolated from humans possess the *vacA* gene, the ability of these to induce cellular vacuolization differs significantly from one strain to another. The polymorphism within the *vacA* gene includes three regions of high sequence diversity and at least two primary variants in each region—the signal, intermediate, and middle regions, which are closely associated with vacuolating activity [18,19]. The signal and middle regions correspond to the two main polymorphic regions and have been characterized as markers of *H. pylori* virulence and the risk of progression to severe diseases [18,19,20].

According to the latest available statistics, in 2020, more than one million new cases of gastric cancer were diagnosed worldwide, accounting for 5.6% of all cancer cases, making it the fifth most diagnosed cancer and the fourth leading cause of death, with around 769,000 deaths per year globally (7.7% of total cases) [21,22]. It is important to note that incidence and mortality rates vary by geographic region, with higher incidence in Asia, Central and Eastern Europe, and South America, compared to the rest of the world [21,22]. This geographic disparity may be due to various factors, such as differences in diet, genetic factors, and limited access to preventive healthcare and early detection programs. In many of these regions, patients with gastric cancer are diagnosed at advanced stages of the disease, resulting in lower survival rates. Among South American countries, Chile has one of the highest age-standardized incidence and mortality rates compared to neighboring countries, with 13.1 and 10.0 per 100,000 inhabitants, respectively [23].

Disparities in the available data highlight an existing gap between the observed incidence of *H. pylori* in different regions and the genotype of the involved bacterial strains, particularly in Latin America. The data of many studies are based on serological tests, although some have included urease and microscopic analyses of biopsies while others have simply used a PCR assay. These approaches represent limitations for comparing and extrapolating some conclusions. Therefore, one purpose of the present review is to motivate other researchers to consider the genetic variants of *H. pylori* as new biomarkers for risk assessments, especially in areas with demonstrated genetic variety, as in South America. 

This review aims to perform a comprehensive analysis of all *H. pylori* populations and subpopulations worldwide. The way in which these genes have spread among various human populations is described, adapting and predominating depending on the geographical location. The predominant genotypes are addressed globally, in Latin America and specifically in Chile, with the aim of understanding the genetic diversity and pathogenic capacity of *H. pylori* in populations with a high risk of gastric cancer.

## 2. Prehistoric Perspectives on the Presence of *H. pylori* in the American Continent and Latin America

### 2.1. Genotypes and Pathogenic Polymorphisms

*H. pylori* has infected the human stomach for at least 88,000–116,000 years [24] and has coevolved with our ancestors during the early human migrations from East Africa approximately 60,000 years ago [25].

Sequencing fragments of seven constitutive genes (*atpA*, *efp*, *mutY*, *ppa*, *trpC*, *ureI*, *yphC*) and a virulence-associated gene (*vacA*) from 370 *H. pylori* isolates from 27 human geographical groups revealed 3850 nucleotides sequenced per strain, of which 1418 nucleotides were polymorphisms used to identify different bacterial populations [26]. Analysis of the genetic structure identified four modern populations, hpAfrica1, hpAfrica2, hpEastAsia, and hpEuropa, based on current distributions [26]. Subsequent analyses, using the same 1418 polymorphisms, divided hpEastAsia into three subpopulations, known as hspAmerind, hspEAsia, and hspMaori, and the hpAfrica1 variant was divided into hspWAfrica (west) and hspSAfrica (south) [26]. Furthermore, 74 additional polymorphisms were found in the same evaluated genes and were present in the three subpopulations derived from hpEastAsia. Of these 74 new polymorphisms, hspMaori shares only between 36 and 39 with the hspEAsia and hspAmerind variants; however, 101 polymorphisms have been described as common between hspEAsia and hspAmerind [26]. Notably, 97 polymorphisms have been reported as exclusive to the hspMaori strains, specifically within the constitutive genes, while approximately 148–167 polymorphisms would be characteristic of the hspEAsia and hspAmerind strains for those same regions [26].

Another study, which included 769 *H. pylori* isolates from 51 different ethnic origins, used sequencing of a 3406-base-pair (bp) fragment covering the constitutive genes *atpA*, *efp*, *mutY*, *ppa*, *trpC*, *ureI*, and *yphC* and revealed the presence of 1522 polymorphic nucleotides, which were used for the analysis of genetic diversity distribution of the same microbial isolates. Phylogenetic analysis identified four previously assigned populations as hpEuropa, hpEastAsia, hpAfrica1, and hpAfrica2, and two new populations, hpAsia2 and hpNEAfrica (north-east), were identified [25]. The population designated as hpAsia2 was isolated in Northern India, Thailand, Bangladesh, the Philippines, and Southeast Asia, while hpNEAfrica was predominant among isolates from Ethiopia, Somalia, Sudan, and Northern Nigeria [25]. Following a similar methodology, Moodley and colleagues, from 212 *H. pylori* isolates obtained from gastric biopsies collected from native inhabitants of Taiwan and Australia, highlanders in New Guinea, as well as Melanesians and Polynesians in New Caledonia, and an additional 100 isolates from Europeans in Australia, sequenced fragments of the previously described constitutive genes (*atpA*, *efp*, *mutY*, *ppa*, *trpC*, *ureI*, *yphC*), covering 3406 bp with 1695 bp being polymorphic. From these isolates, 196 unique haplotypes were obtained, which were compared with 99 unique haplotypes from Europeans in Australia and 222 unique haplotypes from inhabitants of Asia and the Pacific, and according to Bayesian assignment analysis, the samples from the native inhabitants yielded 50 unique haplotypes that formed a distinct biogeographic group, termed hpSahul, where 28% (26 out of 92) of the haplotypes were from Australian Aborigines and 89% (24 out of 27) were from New Guinea highlanders [27].

**Figure 1:**
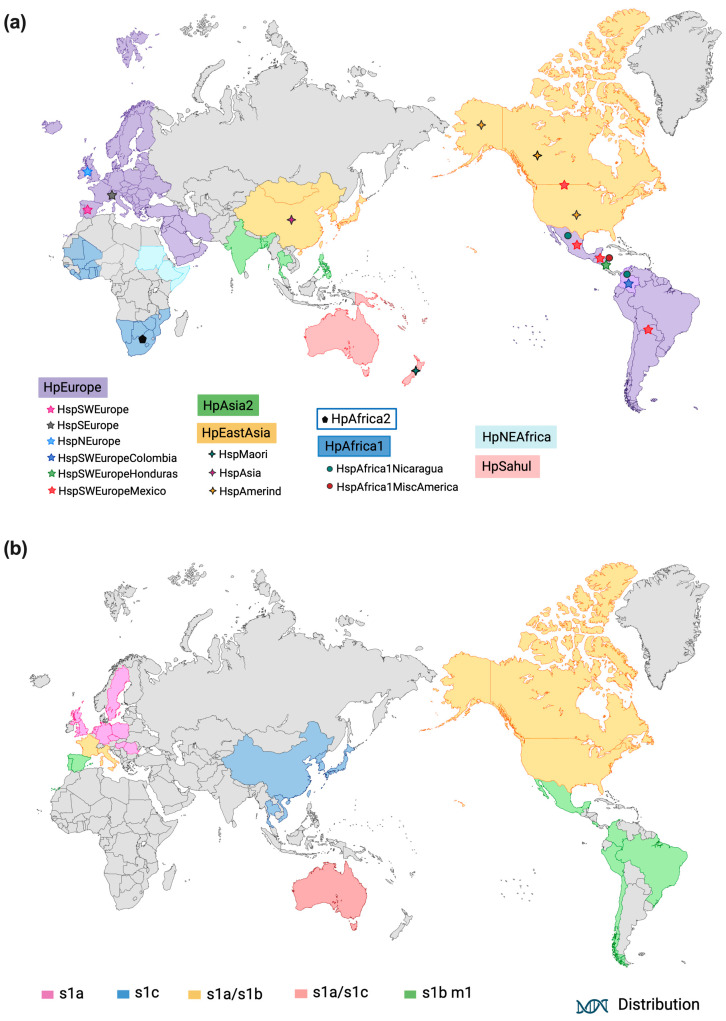
The distribution of *Helicobacter pylori* worldwide: (**a**) *H. pylori* populations and subpopulation; (**b**) *H. pylori vacA* variants. This image was created at BioRender.com. 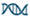: corresponds to the polymorphic variant distribution.

Therefore, the study of seven constitutive genes and one virulence gene from microbial isolates from different regions of the world indicates that *H. pylori* can be classified into seven populations: hpEurope (isolated from Europe, the Middle East, India, and Iran), hpNEAfrica (isolated in Northeast Africa), hpAfrica1 (isolated from West African and South African countries), hpAfrica2 (isolated from South Africa), hpAsia2 (isolated from Northern India and among strains from Bangladesh, Thailand, and Malaysia), hpSahul (Australian Aborigines and New Guinea), and hpEastAsia, with subpopulations hspEAsia (East Asians), hspmaori (Taiwanese Aborigines, Melanesians, and Polynesians), and hspAmerind (Native Americans) (Figure 1a, and Table 1) [25,26,27].

All these modern populations derived from six ancestral populations were designated as ancestral Europe1 (AE1), ancestral Europe2 (AE2), ancestral East Asia, ancestral Africa1, ancestral Africa2, and ancestral Sahul [25,26,27].

In addition to constitutive genes as a phylogenetic indicator, it has been reported that virulence genes also contribute to the genetic and migratory evolution of *H. pylori*. The *vacA* contains a region encoding the signal peptide with four allelic subtypes, s1a, s1b, s1c, and s2, and another middle region, which has two allelic types, m1 and m2, of which m2 presents two subtypes, m2a and m2b [18,38]. The *cagA* is highly polymorphic in the 3’ region, due to the presence of the EPIYA motif (Glu, Pro, Ile, Tyr, Ala) in the C-terminal region of the protein, which generates different patterns in the carboxy-terminal of the protein. The four different types of EPIYA motifs, EPIYA-A, -B, -C, and -D, are determined by the surrounding amino acid sequence, with the EPIYA-A and EPIYA-B motifs being representative polymorphisms shared by almost all *H. pylori* isolates, followed by the EPIYA-C or EPIYA-D motifs [11]. Based on the expression of these two virulence factors, *H. pylori* strains have been classified into three groups, i.e., with higher virulence potential, *cagA*+/*vacA*s1 (type I), and with lower virulence, *cagA*−/*vacA*s2 (type II), and strains with an intermediate virulence genotype, *cagA*−/*vacA*s1 or *cagA*+/*vacA*s2 (type III) [39]. On the other hand, the 3’ end of the *cagPAI* has five types of deletion, insertion, and substitution motifs that allow studies of the different *H. pylori* genotypes distributed geographically and the possible evolutionary origins [28].

### 2.2. Global Molecular and Geographical Evolution

The analysis of the geographic distribution of *vacA* allelic types in 735 *H. pylori* strains isolated from patients from 24 countries showed that 611 strains have a unique genotype. This analysis identified that 89% of the strains with the s1a subtype were found in Northern and Eastern Europe and in the region spanning Spain and Portugal. In France and Italy, the s1a and s1b subtypes were present in the same proportions (47.5% and 52.5%, respectively). In Central and South America, all strains were classified as subtype s1b, except in North America where there was an equal proportion of s1a and s1b subtypes (48.8% and 51.2%, respectively). On the other hand, in East Asia, the s1c subtype was dominant, encompassing 77% (57/70) of the strains analyzed in that region. Regarding the middle region of vacA, the m1 subtype was most prevalent, with 86.7% in Spain, Portugal, Central, and South America, and the m2b subtype was found only in s1c strains from East Asia (Figure 1b) [29].

Studies conducted on 500 *H. pylori* strains isolated from five different geographical regions, including Latin America, Europe, Africa, the United States, and Asia, revealed five types of deletion, insertion, and substitution at the right end of the *cagPAI.* These changes primarily involved remnants of a transposable element (insertion sequence, IS) termed IS606*, a second small transposable element called miniIS605, a small helicase gene (*hel*), and small DNA segments with no homology to sequences available in databases. Five types of DNA motifs were identified based on (i) the lengths of IS606* (approximately 130 bp in types I and III, 312 bp in type II, 35 bp in type IV, and 124 bp in type V); (ii) the lengths of the *hel* gene (approximately 423 bp in type I, 9 bp in type II, and 896 bp in type IV); (iii) various other sequences, either between the remnants of the IS606* gene and *hel* gene or replacing the *hel* gene; (iv) the presence or absence of the miniIS605 element within the IS606*; and (v) in some cases, the presence of full-length IS606. Type II strains were also distinguished by a specific 22 bp deletion immediately following the *hel* gene sequences, which was not found in other strains. Categorization of strains based on the three most common domains established that type I was most predominant in Spaniards, native Peruvians, Guatemalan Ladinos (mixed Amerindian-European descent), and native Africans. On the other hand, type II predominated among Japanese and Chinese, and type III in Kolkata (India). The type IV motif is rare and was found in a single strain isolated in the United Kingdom and two strains from West Virginia (USA). The type V motif has been found in some strains from India, specifically from the Kolkata region [28].

Regarding the observations in Latin American isolates, the sequences in the *cagA* gene and the *vacA* m1-type alleles from strains obtained from native Peruvians show high similarities with the sequences obtained from microbial isolates in the Spanish, compared to those isolated from Asian individuals. This kinship in Latin American and Spanish strains has led to the hypothesis that *H. pylori* may have been brought over by European conquerors and settlers about 500 years ago [28], suggesting that different types of *H. pylori* virulence genes are associated with different human ethnic groups [28,30,40]. Specifically, *vacA* s1a and s1c are predominant in Northern Europe and Asia, respectively, while s1b is common in South America, Southern Europe, and South Africa (Figure 1b) [29,41,42].

### 2.3. Arrival and Evolution of H. pylori in Latin America

The rapid evolution of *H. pylori* since European colonization has influenced the differentiation of *H. pylori* subpopulations among the countries of the Southern American cone [32,43]. This evolution has even led to the identification of a variant of the *BabA* gene (adhesin binding to blood group antigens), now considered an exclusive Latin American descendant [33].

Genetic studies conducted on *H. pylori* isolates from natives residing in Shimaa, an Amerindian village in Peru, have demonstrated that the Asian ancestry is preserved from the migration of humans to America via the Bering Strait [30,31]. These isolates generally belong to the hspAmerind subgroup of hpEastAsia, reflecting the ancestral human movement from Asia to South America [31]. Specifically, the sequencing and phylogenetic analysis of constitutive genes of 44 strains linked the *H. pylori* isolates from the Peruvian Amazon with strains from Japan. This study confirmed that the 44 strains contained a single s1 allele, at least three polymorphisms in the middle region of the *vacA* gene, specifically m1b (29 cases), m2 (13 cases), and m1b/m2 (2 isolates), as well as a new allele of the *hp0519* gene [31]. 

A study conducted on 400 *H. pylori* isolates from 20 patients in Mexico City allowed for the characterization of the *vacA* and *cagA* genotypes [34]. The results showed that the patients were infected with multiple strains with different *vacA* genotypes; specifically, 17 patients presented strains of *H. pylori* with two or more genotypes, the most frequent being the s1b m1 genotype, and in 5 patients, strains of *vacA* type s2 m1 were found, which had not been previously described. All patients were infected with *cagA*-positive strains, but these occasionally coexisted with a small number of *cagA*-negative strains [34]. According to the authors’ conclusions, coinfection with multiple strains of *H. pylori* is common in Mexico, and *vacA* in these strains is genetically more diverse than has been described in other populations. 

Phylogenetic analysis of *H. pylori* from 35 native participants in Mexico, including 18 cases of Nahuas, 11 of Tarahumaras, 5 of Huicholes, and 1 single case from Otomi, showed that the majority of the *vacA* strains contained the European s1b m1 subtype (13 Nahua and 7 Tarahumara), and 6 isolates were s1b m2 (4 Huicholes, 1 Tarahumara, and 1 Otomi), and some were of the s2 m2 type (4 Nahua and 3 Tarahumara), and the only the Huichol isolate (368H) contained the allele s1c [30]. In addition, 31 of the isolates carried the virulence gene *cagA*, with only three EPIYA-type repeats and a multimerization motif; in 10 strains, the EPIYT-B-type variants and/or multimerization motif variants (more frequently lysine at position 5) were found; in the Tarahumara strains, two multimerization motifs were found, and the 23O (Otomi) and 368H strains showed a variant GSIYD B motif and a partially deleted multimerization motif [30]. Additionally, phylogenetic analyses of *cagA* and *vacA* showed that most of the isolates from Mexican natives were in the Western group, except the Huichol 368H and Otomi 23O strains, which were grouped in a group related to the East Asian group [30]. On the other hand, the phylogenetic analysis of *hspA* showed that the isolates from the three Mexican indigenous groups (Nahuas, Tarahumaras, Huicholes) were present in all previously described Asian, European, and African groups, and therefore, the autochthonous strains (368H and 23O) are grouped within the Asian group [30].

To analyze the population structure of *H. pylori* in mestizo individuals in Latin America, a study analyzed the genome of 107 *H. pylori* strains isolated from biopsies of patients in Mexico, Nicaragua, and Colombia, comparing them against 59 publicly available genomes [32]. The results of the phylogenetic analyses focused on the *cagPAI* gene and revealed two main groups in the Latin American population. The first, called LAmerica (1), is composed of isolates from Mexico, Colombia, and Nicaragua, and a second group, LAmerica (2), which includes strains from Nicaragua, Colombia, Mexico, and El Salvador. A specific group formed only by Colombian strains, called the Colombia group, was also identified. Additionally, analyses of the *cagA* gene confirmed the separation of Latin American strains from those found in hpEurope, hpAsia, and hspAmerind [32]. Therefore, the authors conclude that *H. pylori* populations in Latin America have rapidly evolved to adapt to the different human groups present in this region [32].

Recently, analysis of the genome of *H. pylori* from 723 strains isolated from 14 countries in America, as well as strains from the Iberian Peninsula and public genomes available in databases from Europe, Africa, and Asia, showed distinct genomic footprints for each studied region and revealed new subpopulations of *H. pylori* [35]. Specifically, analyses of genomic ancestry using data from 498,461 single nucleotide polymorphisms in 1385 genes allowed the identification of new subpopulations across the continent, including a new European subpopulation called hspSWEurope, which includes strains isolated in the Iberian Peninsula (Spain and Portugal), distinct from the subpopulation hspSEurope (Belgium, Germany, Italy, France, and some places in South Asia). These were also distinct from hspNEurope, which includes mostly isolates from Sweden, the UK, and Ireland (Figure 1a). Additionally, three subpopulations with European ancestry were identified, called hspSWEuropeColombia (isolates from Colombia), hspSWEuropeHonduras (isolates from Honduras, Nicaragua, Guatemala, and El Salvador), and hspSWEuropeMexico (isolates from Mexico, and North, Central, and South America), and two subpopulations with African ancestry, hspAfrica1Nicaragua (isolates from Nicaragua and Honduras) and hspAfrica1MiscAmerica (isolates from Mexico and Colombia) [35] (Figure 1a). Additionally, fixation-index analyses on the complete genome identified common genetic variants in America, showing 142 sites with significant fixation-index values in 35 genes, of which 22 encode virulence factors and membrane proteins, and 15 of the 35 genes were present in all the strains studied, including *vacA*, *hofC*, *ppiC*, another three membrane proteins, fecA, and a Zn-dependent protease. These findings suggested that virulence plays an important role in adaptation to specific human populations [35]. The variability in virulence genes was strongest in America, and much of the variation corresponded to nonsynonymous substitutions in functional domains of the main virulence proteins (CagA, VacA, and BabA). In particular, six nonsynonymous substitutions were identified in cagA—four in domain II and two in domain III, nine amino acid nonsynonymous substitutions in vacA, located in the middle region of the protein, and three nonsynonymous substitutions in amino acids 49, 53, and 153 of BabA. Apparently, these virulence genes have followed unique evolutionary paths in American populations, potentially contributing to the high risk of gastric cancer in the region [35].

A recent study based on the genome analysis of 1011 clinical strains of *H. pylori* from the *Helicobacter pylori* Genome Project, collected from 50 countries worldwide, has provided detailed insights into core genomic diversity and population structure. For the analysis, 255 reference genomes from diverse regions worldwide were included, enabling an outlining of ancestral contributions to Eurasian, African, and American populations [44]. The results revealed a new subpopulation, hspEurasia, that includes the hspCEurope/hspSEurope and hspMiddleEast subpopulations. This subpopulation included hspEurasia1 in Central and Eastern Europe, featuring genomes from strains in Germany, Poland, Lithuania, Latvia, Turkey, and Russia; hspSouthern Eurasia2 with strains from France, Italy, Greece, Jordan, and Iran; and hspEurasia3 with strains from India, Bangladesh, and Greece. The study observed that European subpopulations exhibit varying proportions of ancestry, with an increase in Asian ancestry and a decrease in African ancestry in the hspEurasia1 and hspNEurope populations. The hspSWEurope population showed a higher proportion of hspAfrica1Wafrica ancestry, with a similar distribution of hpNEAfrica. Furthermore, hspEurasia3 presented a more marked ancestry from hpAsia2 compared to the other hpEurope populations, and hspUraI (subpopulation of hpAsia2) was the most prominent Asian ancestor [44]. Regarding African genomes, the analyses showed that *H. pylori* from North Africa was more similar to Iberian and Middle Eastern bacteria than to African bacteria. The analysis of 238 strains from different regions of Latin America showed that the majority were grouped into the populations hspAfrica1MiscAmerica, hspSWEuropeLatinAmerica, and hspSWEuropeChile. Approximately a third of the genomes were clustered into hspAfrica1Safrica and hspSWEurope. The hspEurasia subpopulation was also identified in Argentina, Brazil, and Chile, along with two hspEurasia genomes in Brazil. In general, Latin American genomes displayed greater admixture than their European and African counterparts. Furthermore, these genomes confirmed the existence of an hspIndigenousAmerica population, which includes the hspIndigenousNAmerica subpopulation with strains from indigenous communities in North America (Canada and the USA), and the hspIndigenousSAmerica subpopulation with isolates from Latin America. Through the analysis of 68 *H. pylori* genomes from the United States, the study identified a highly clonal but geographically dispersed North American subpopulation, which lacks the cag pathogenicity island and is present in 7% of the sequenced genomes [44].

## 3. Molecular and Epidemiological Scenario of *H. pylori* and Gastric Cancer in Chile

### 3.1. Local Genetic Variants of H. pylori

Previously, from 63 *H. pylori* strains isolated from Chilean patients without differentiation of geographical location, the *cagA* gene and the s1 m1 allelic variants of the *vacA* gene had a prevalence of 52%, being more common in patients with peptic ulcers than in strains isolated from patients with non-ulcer dyspepsia (26%) (*p* = 0.035) [45]. Similarly, a study of the *cagA* and *vacA* genes of *H. pylori* strains isolated from 50 Chilean patients with gastrointestinal symptoms revealed the presence of *cagA* in 19 samples (38%). The prevalence of the s1 and s2 signal sequences of *vacA* was similar, being detected in 16 samples each (32%), and the m2 middle region predominated in 29 samples (58%). In patients where only one *vacA* genotype was detected, the most frequent was s2 m2, followed by s1 m1 and s1 m2, and one strain was detected with the s2 m1 genotype [46]. Studies conducted from 245 *H. pylori* isolates obtained from biopsies of 79 patients from different places in Chile (Iquique, La Calera, Quillota, Valparaíso, Santiago, Linares, Los Angeles, Temuco, Valdivia, and Punta Arenas) and who suffered from gastrointestinal diseases revealed that the most prevalent *vacA* variant corresponds to the s1b m1 genotype (72%), followed by s1a m1 (25%), and, sparsely, s2 m2 (3%) [36]. The prevalence of these variants showed a differentiated geographical distribution. Patients north of Santiago exclusively carry s1b m1, while patients from Santiago and south of Santiago would carry strains with one or both genotypes (s1a m1 and s1b m1) [36]. 

Another study analyzed 66 isolates of *H. pylori* obtained from 41 patients in Concepción, who consulted due to suspicions of upper digestive pathology between 2001 and 2002. The presence of *cagA*, *vacA* (s and m), and *babA2* genes was determined. The results showed the detection of the *cagA* gene in 24.2% of the clinical isolates analyzed (16/66), while the *vacA* s1a gene was detected in 42.4% of the samples (28/66), *vacA* s1b in 21.2% (14/66), *vacA* s2 in 25.8% (17/66), *vacA* m1 in 31.8% (21/66), and *vacA* m2 in 43.9% (29/66). It is worth noting that one isolate (1.5%) was identified that was positive for the babA2 gene, constituting the first record of this genotype in Chile. Furthermore, this clinical isolate also carried the *cagA*+ and *vacA* s1a genes that have been linked to severe gastric diseases, and there were five isolates that showed an ulcerogenic profile with the *cagA*+ and *vacA* s1 m1 genes [37]. 

On the other hand, the analysis of 78 clinical isolates of *H. pylori* from biopsy samples of antral mucosa from 100 patients with dyspeptic symptoms in the Maule Region, identified the *cagA* gene in 94.9% of all isolates (74/78) and the *vacA* gene in 100% of the isolates (78/78). The s2 and m2 alleles of *vacA* were the most representative (74.4% and 75.6%, respectively), with an association of 58.1% with respect to *cagA*-positive isolates. Additionally, it was determined that 48.6% of the *cagA*-positive strains harbored two or more EPIYA-C motifs, which were associated with more severe pathological findings [47].

In this sense, a previous study that involved the isolation of 38 strains from patients with non-ulcer dyspepsia and 25 from individuals with peptic ulcer showed that cagA was present in 60% of the patients with peptic ulcer (15 out of 25) and in 55% of the patients with dyspepsia (21 out of 38). The prevalence of the s1 allelic variant of *vacA* was similar among strains obtained from ulcers or patients with non-ulcer dyspepsia. However, the *cagA*+/*vacA* s1 m1 combination was found more frequently among *H. pylori* strains from patients with peptic ulcer (52%) than among strains isolated in the other group (26%) (*p* = 0.035). The authors concluded that the presence of *cagA* or the s1 *vacA* allelic variant alone would not have predictive value as a risk marker for serious gastric pathologies in the Chilean population. However, being infected with an *H. pylori* strain with the *cagA*+/*vacA* s1 m1 genotype could be associated with a higher risk of acquiring peptic ulcer disease [45].

This suggests that the s1 m1 alleles of the *vacA* gene and *cagA*-positive strains are representative in Chilean patients and, given the limited literature, it is possible to infer that these genes are related to more serious gastrointestinal pathologies. Their presence in the population depends on geographical distribution, and it could be hypothesized that strains with these genotypes would be of European ancestry.

### 3.2. Epidemiological Retrospective of H. pylori and Gastric Cancer

While the genotypes present globally, in Latin America, and Chile have been described, it is important to understand the prevalence of *H. pylori* in Chile and its correlation with a higher risk of gastric disease. One of the first studies to determine the prevalence of *H. pylori* was conducted on symptomatic and asymptomatic individuals of low socioeconomic status in Santiago and was published in 1993 [48]. The endoscopic and histological examination of 111 cases demonstrated changes in the gastric mucosa in 91% of the patients, with the most prevalent conditions being gastritis (71%) and duodenal ulcer (20%). The observed prevalence of *H. pylori* in biopsies of patients with gastritis was 90%, and for those with duodenal ulcers, it was 100%. In individuals with normal gastric findings, it was 3 out of 10 (30%, *p* < 0.01) [48]. Subsequently, a study that included 190 control individuals and 236 patients with varying degrees of endoscopic esophagitis (55 with gastroesophageal reflux, 81 with erosive esophagitis, and 100 with Barrett’s esophagus) determined a similar prevalence of *H. pylori* (20–32%) in the gastric antrum of healthy controls and in any group of patients with endoscopic esophagitis [49]. No differences were observed in distribution by age and sex, and the infection of *H. pylori* in the gastric fundus was less than 5% [49].

On the other hand, the evaluation of *H. pylori* in 200 patients with chronic gastritis from the Temuco hospital showed that 82% had *H. pylori*-like organisms. Additionally, the infection was present in 92.7% of patients with gastric ulcers and in 94.4% of patients with duodenal ulcers [50]. The prevalence of *H. pylori* infection in 276 patients from the Metropolitan Region showed that the bacteria were detected using the urease test in 124 patients (44.9%), and the infection was more prevalent in younger patients (53.8%, between 21 and 60 years) and lower in patients over 60 years (25.6%) [51]. 

The largest study conducted in Chile, reported in 2010, included 5664 symptomatic patients, of which 59.3% presented a normal endoscopic diagnosis, 20% had erosive esophagitis, 8.1% gastric ulcer, 6.4% duodenal ulcer, and 6.2% erosive gastropathy. Of the cases, 78% were infected with *H. pylori*, and the prevalence of infection was higher in patients with duodenal ulcers (86.6%, *p* < 0.001), followed by gastric ulcers (81.4%, *p* = 0.02), erosive gastropathy (79.9%), and erosive esophagitis (77%). The authors concluded that the prevalence of *H. pylori* infection is high in symptomatic Chilean patients and even higher in those with gastroduodenal ulcers or erosions, while in patients with erosive esophagitis, the incidence is similar to those with a normal endoscopy [52].

Moreover, the prevalence of *H. pylori* infection in 274 pregnant Chilean women from the Biobío Region, and its relationship with gastrointestinal symptoms, showed that 68.6% had *H. pylori* infection (188 of 274), with no differences between the population with symptoms of dyspepsia, and only 63 women (23%) were negative [53]. More recently, an innovative study demonstrated a prevalence of 76.9% for *H. pylori* and 25.4% for the *cagA*strain from stool analysis in 160 symptomatic patients from the Coquimbo Region, with no significant differences between genders [54]. More recently still, a cross-sectional study conducted between January and December 2018 gathered a total of 229 antral biopsies from dyspeptic patients undergoing endoscopy in the public (n = 143) and private (n = 86) health systems, all from the Araucanía Region, with an observed prevalence of *H. pylori* of 45.41%, with infection risk showing a higher association with the Mapuche ethnicity (OR = 2.30, CI = 1.14–4.78, *p* = 0.026) [55]. These results were later validated in an independent cohort of 155 cases with similar clinical and demographic characteristics, verifying a prevalence of *H. pylori* in the general population of 43.2% (67 of 155) and 55.0% (22 of 40) within the Mapuche population [56].

Based on all the aforementioned data, the prevalence of *H. pylori* varies between 20% and 92% (Table 2). Its relationship with an increased risk of gastrointestinal disease has been mentioned; however, the determination of the bacteria has been developed by different methods that in some cases cannot be comparable and vary significantly in the detection percentage. Through an ELISA assay, serum IgG antibodies against *H. pylori* were determined in 2615 individuals, and the results showed a prevalence of *H. pylori* of 73.0% (95% CI, 70.0–76.0%), with higher infection in men from 45 to 64 years old, decreasing after 65 years old [57]. Using a hierarchical Poisson regression model, the spatial distribution of deaths from gastric cancer between 1985 and 2002 in 333 municipalities was analyzed, classified into low, medium, and high mortality, and showed mortality rates of 11.4, 19.1, and 26.0 per 100,000 inhabitants, respectively. It is noteworthy that the prevalence of *H. pylori* was higher among residents of places with high mortality from gastric cancer (79.7%; 95% CI, 76.4–82.6) compared to places with low gastric cancer mortality (62.3%; 95% CI, 53.8–70.2; corresponding proportional reporting ratio, 1.3; 95% CI, 1.1–1.5), according to the regression model used [57].

Another study allowed the evaluation of the difference in the detection of premalignant gastric lesions using the Sydney protocol (n = 124) compared to the conventional endoscopic approach (n = 146). In relation to the result of the biopsies, it was found that 49.2% of the biopsies performed with the Sydney protocol were positive for the presence of *H. pylori*, in contrast to 20.5% of the biopsies without the protocol (*p* < 0.001). The authors concluded that the Sydney protocol significantly increases the detection of *H. pylori* infection and the differential diagnosis of the premalignant cascade compared to the study without a protocol [58]. Subsequently, a study considering the population of Temuco conducted on 485 endoscopic biopsies under the OLGA criterion established a prevalence of *H. pylori* of 30.3% of patients (150 of 485) using methylene blue histological staining and differential Quik staining (Diff-Quik), with a higher incidence in men (60%) than women (40%) (*p* < 0.001). Additionally, infection with *H. pylori* was greater in younger groups (45–56 years, *p* < 0.05), and those patients who were infected with *H. pylori* had more gastric atrophy and metaplasia than those without infection (*p* < 0.05) [59]. 

To date, one of the most significant studies corresponds to the comparison of two locations with extreme differences in incidence and mortality associated with gastric cancer. The Epidemiological Investigation of Gastric Malignancies study compared the cities of Antofagasta and Valdivia, with the lowest and highest rates of gastric cancer, respectively. The incidence of *H. pylori* observed in 700 participants was 67% (95% CI = 63–71) in Antofagasta (lower risk gastric cancer area) and 63% (95% CI = 60–67) in Valdivia (higher risk gastric cancer area). The age-adjusted prevalence was 58% (95% CI = 51–69) in Antofagasta and 56% (95% CI = 49–66) in Valdivia. The seroprevalence of *H. pylori* was 20% among children < 10 years, 40% among those 10 to 19 years old, 60% among those 20 to 29 years old, and close to or over 80% in those over 30 years old. Therefore, the prevalence of *H. pylori* infection and its virulence factors was similar in the high- and low-risk areas, but atrophy was more common and occurred at younger ages in the higher-risk areas. Surprisingly, from a multivariate model that combined both sites, age, spicy food consumption, and the presence of *cagA* were the main risk factors for gastric atrophy, with dietary factors being the most determinant in the rates of gastric atrophy, acting as the dietetic factors most determinant in the rate of atrophy and gastric cancer among the national population [60].

## 4. Conclusions

*H. pylori* is a bacterium that colonizes the gastric mucosa in over 50% of the world’s population and is responsible for triggering a series of gastrointestinal problems. Infection with *H. pylori* strains that are positive for the *cagA* gene significantly increases the risk of gastric cancer due to more severe inflammation and cellular damage compared to strains lacking the *cagA* gene. Globally, its prevalence varies depending on the geographic region, being higher in Asia than in Western countries. On the other hand, the VacA protein is an intracellularly acting exotoxin that affects multiple cellular pathways in the host, inducing vacuolization and cell death. Although most *H. pylori* strains have the *vacA* gene, the ability to induce cellular vacuolization varies among different strains of the bacterium. Worldwide, *cagA*-positive strains are responsible for the largest proportion of *H. pylori* infections in individuals, and this protein is involved in the development of more severe premalignant lesions of the gastric epithelium [61]. As presented above, the polymorphic variants of *cagA* have allowed new epidemiological classifications of the infected population; however, after the gene is expressed, structural differences between CagA proteins (or ethnic isoforms) may explain the discordant information in the incidence of gastric cancer for some human groups. 

*H. pylori* strains in regions excluding East Asian countries contain the CagA protein with EPIYA segments arranged as EPIYA-A (32 amino acids), EPIYA-B (40 amino acids), and EPIYA-C (34 amino acids) and is, therefore, referred to as Western CagA or ABC-type CagA [62]. The EPIYA-C segment presents in a variable number of copies among distinct Western CagA variants, typically represented in tandem between one to three times [62]. The EPIYA-repeat region of CagA found in East Asian countries also possesses EPIYA-A and EPIYA-B segments but, instead of the tandem EPIYA-C segment, contains a distinct EPIYA-containing segment termed EPIYA-D (47 amino acids), and the CagA protein is referred to as East Asian CagA or ABD-type CagA [11]. In turn, the Amerindian CagA protein seems to have a hybrid segment combining a part from the original EPIYA-C and EPIYA-D plus a non-canonical CM segment that is unique compared to the other two isoproteins [63]. Reports state that EPIYA-A and EPIYA-C or EPIYA-B and EPIYA-D are preferably phosphorylated in combination in Western CagA and East Asian CagA, respectively [64], thus promoting intracellular signaling with multiple deleterious effects on the gastric epithelium. However, the biochemical and physiological implications of these major differences in the Amerindian CagA have not yet been deeply studied, leaving an unexplored niche for further investigations to explain the pathogenic role of this Amerindian CagA-positive strain in the Latin American population.

Several studies have demonstrated that *H. pylori* affects immune infiltration, mainly by impairing proliferation or promoting apoptosis in T-cells [65,66,67]. In addition, gastric epithelial cells constitutively express class II Major Histocompatibility Complex molecules and may display a role as secondary antigen-presenting cells; however, the VacA toxin can impair the presentation of the Major Histocompatibility Complex-antigen [68]. Furthermore, *H. pylori* can induce the expression of PD-L1 in infected gastric epithelial cells, which is a negative immune checkpoint molecule able to induce the apoptosis of T-cells [69,70]. These findings represent a mechanism of immune evasion in favor of bacterial infection. Altogether, this may be used to shape the gastric-tumor microenvironment, where the presence of lymphocytes may be negatively regulated by the presence of any *H. pylori* strain (or polymorphic variant), eventually limiting the required cytotoxic activity to eradicate tumor cells within the tumor microenvironment. Unfortunately, not much data exist to date to address this hypothesis, and new studies will be needed to correlate the polymorphic variants of *H. pylori* with the outcome of gastric cancer patients under any therapeutic regimen, in particular those based on immunotherapy.

More than one million new cases of gastric cancer were diagnosed worldwide in 2020, making it the fifth most common cancer and the fourth leading cause of cancer mortality. Incidence and mortality vary by geographic region, with higher rates in Asia, Central and Eastern Europe, and South America due to differences in diet, genetic factors, and limited access to preventive medical care. Chile has one of the highest age-standardized mortality rates compared to neighboring countries.

Phylogenetic analyses of the polymorphisms of constitutive and virulence genes in *H. pylori* have classified it into populations and subpopulations depending on their geographical distribution in strains hpAfrica1 (subpopulations hspWAfrica and hspSAfrica), hpAfrica2, hpEastAsia (subpopulations hspAmerind, hspEAsia, and hspMaori), hpEuropa, hpAsia2, hpNEAfrica, and hpSahul, derived from six ancestral populations. Additionally, *H. pylori* strains have been classified into three groups according to the expression of virulence factors: type I strains that are highly virulent (*cagA*+/*vacA*s1), type II strains that are less virulent (*cagA*−/*vacA*s2), and type III strains with intermediate virulence (*cagA*−/*vacA*s1 or *cagA*+/*vacA*s2). This classification helps to understand the genetic diversity and pathogenicity of *H. pylori* in different populations and geographic regions. The high variability in the s1a, s1b, and s1c subtypes of the *vacA* gene of *H. pylori* in different regions has determined geographic distribution, showing a higher prevalence of s1a and s1b in Northern and Eastern Europe, s1b in Central and South America, and s1c in East Asia. The high sequence similarity in the virulence genes between *H. pylori* strains from Peruvian natives and Spaniards in Latin America suggests a possible European influence, and the most recent analyses have revealed new genetic subpopulations of *H. pylori* in different regions of America and Europe, suggesting adaptations to human populations.

In Chile, *H. pylori* strains that are *cagA* positive and possess the allelic variant s1 m1 of the *vacA* gene are more frequent in patients with gastrointestinal diseases. Furthermore, the presence of these strains varies significantly according to the geographical location of the Chilean population. The prevalence of *H. pylori* in Chile ranges from 20% to 92%, and this variation is directly related to geographic distribution. In places where the prevalence of *H. pylori* is higher, there is a greater proportion of patients with more severe gastrointestinal diseases. However, it is important to note that the distribution of *H. pylori* in the Chilean population and its possible correlation with more severe gastrointestinal pathologies, such as the risk of developing gastric cancer, cannot be completely comparable, mainly due to differences in the techniques used to determine the presence of the bacterium in the population.

## Figures and Tables

**Table 1:** The populations and subpopulations of *Helicobacter pylori* depending on their geographical location.

Analyzed Strains	Included Countries	Target Genes	Main Findings	Ref.
769	Japan, Korea, China, Taiwan, Vietnam, Singapore, Malaysia, Thailand, Philippines, Ladakh, Bangladesh, Kazakhstan, New Zealand, Samoa, Australia, Canada, USA, Colombia, Venezuela, Peru, Estonia, Finland, Russia, Germany, Italy, Netherlands, Spain, UK, Turkey, Jordan, Lebanon, Israel, Egypt, Nigeria, Sudan, Ethiopia, Somalia, Algeria, Morocco, Senegal, Burkina Faso, Southern Africa	*atpA*, *efp*, *mutY*, *ppa*, *trpC*, *ureI*, *yphC*	1522 SNPs Community association hspEAsia; hpAsia2; hpNEAfrica and hpAfrica2; hpEurope	[25]
370	Korea, Singapore, India, South Africa, Burkina Faso, Senegal, Sudan, USA, Canada, Colombia, Venezuela, New Zealand, Australia, UK, Estonia, Finland, Germany, Italy, Spain, and Israel	*atpA*, *efp*, *mutY*, *ppa*, *trpC*, *ureI*, *yphC*, *vacA*	1418 SNPs Community association hspEAsia; hspMaori; hspAmerind; hspSAfrica; hpAfrica2; hspWAfrica; hpEurope	[26]
312	Taiwan, Papua New Guinea, New Caledonia, and Australia	*atpA*, *efp*, *mutY*, *ppa*, *trpC*, *ureI*, *yphC*	1695 SNPs Community association hpSahul	[27]
500	Guatemala, Peru, Spain, Sweden, Lithuania, Gambia, South Africa, Tennessee, West Virginia, Ohio, Missouri, Louisiana, India, Japan, China, Hong Kong	*cagPAI*, *cagA*, *vacA* (allele m1)	cagPAI right-junction motifs Community association Spanish, Native Peruvians, Ladino Guatemalans, Native Africans, Japanese, Chinese, Calcutta, India, UK, West Virginia, USA. cagA and vacA sequence similarity community association Peruvians and Spanish	[28]
735	Australia, Brazil, Canada, Czech Republic, China, Colombia, Costa Rica, Egypt, France, Germany, Hong Kong, Hungary, Italy, Japan, Netherlands, Peru, Poland, Portugal, Romania, Spain, Sweden, Thailand, United Kingdom, USA.	*vacA*, *cagA*	Community association * s1a variant Northern and Eastern Europe s1b variant Central and South America; Spain, Portugal, France, Italy, Canada, and the USA s1c variant East Asia m1 variant Spain, Portugal; North, Central, and South America; Northern and Central Europe, and Australia	[29]
35	Mexico: Tarahumaras, Huichols, Nahuas, and Otomi	*vacA*, *cagA*, *hspA*	Community association s1b m1 variant Nahuas, Tarahuramas s1m2 variant. Huichols, Otomi	[30]
44	Shimaa in the Peruvian Amazon	*atpA*, *recA*, *glmM* (*urec*), *ppa*, *cysS*, *glr* (*murl*), *vacA*, *cagA*, *hp0519*	Community association hspAmerind	[31]
107	Mexico, Nicaragua, and Colombia	*cagPAI*, *cagA*	Phylogenetic analyses of cagPAI Community association LAmerica1: Mexico, Colombia, and Nicaragua. LAmerica2: Nicaragua, Colombia, Mexico, and El Salvador	[32]
52	Nicaragua	*cagA*, *vacA*, *babA*, *sabA*	Whole genome comparison Community association hspWestAfrican and urban South- and Central American. *cagA* and s1/i1/m1 77% of the isolates	[33]
400	Mexico	*vacA*, *cagA*	s1b m1 More frequently	[34]
723	Spain, Portugal, Belgium, Germany, Italy, France, South Asia, Sweden, UK, Ireland, Colombia, Honduras, Nicaragua, Guatemala, Salvador, Mexico, North, Central, and South America	*cagA*, *vacA*, *babA*	498,461 SNP Community association hspSWEurope, hspSWEuropeColombia, hspSWEuropeHonduras, and hspSWEuropeMexico, hspAfrica1Nicaragua, hspAfrica1MiscAmerica	[35]
245	Chile (Iquique, La Calera, Quillota, Valparaíso, Santiago, Linares, Los ángeles, Temuco, Valdivia and Punta arenas)	*vacA*	s1b m1 More frequently	[36]
66	Chile (Concepción)	*cagA*, *vacA*, *babA2*	s1a More frequently	[37]

* Places with more than 50% prevalence are presented.

**Table 2:** Prevalence of *H. pylori* in Chile.

Total Cases	Age (Years)	% Female Group	Diagnostic Tool	Observed Prevalence	Region of Study	Ref.
251	30.5	65.2	Biopsy: microscopy, culture, and urease test	92%	Metropolitan	[48]
426	45.9	57	Biopsy: microscopy, culture, and urease test	20–32%	Unspecified	[49]
200	59.5	48.9	Microscopy alone	82%	Araucania	[50]
276	50.6	70.6	Urease test	44.9%	Metropolitan	[51]
2615	45	52.1	Serology: IgG ELISA test	73%	Unspecified	[57]
5664	50.7	73.1	Urease test	78%	Metropolitan	[52]
100	ND	ND	Biopsy: microscopy, culture, and urease test	78%	Maule	[47]
274	ND	100	Serology: IgG ELISA test	68.6%	Biobio	[53]
270	59	36.3	Microscopy alone	49.2%	Metropolitan	[58]
160	55.6	71.9	Microscopy alone	74.6%	Coquimbo	[54]
229	50.7	64.2	Urease test	45.4	Araucania	[55]
485	54.5	63.1	Microscopy alone	30.3%	Araucania	[59]
155	51.1	66.4	Urease test	43.2%	Araucania	[56]
1395	62.5	ND	Serology: IgG ELISA test	67% and 63%	Antofagasta and Los Ríos	[60]

ND: No data.

## Data Availability

No new data were created or analyzed in this study. Data sharing is not applicable to this article.
